# Excessive β-Catenin in Excitatory Neurons Results in Reduced Social and Increased Repetitive Behaviors and Altered Expression of Multiple Genes Linked to Human Autism

**DOI:** 10.3389/fnsyn.2020.00014

**Published:** 2020-03-31

**Authors:** Jonathan Michael Alexander, Antonella Pirone, Michele H. Jacob

**Affiliations:** Department of Neuroscience, Sackler School of Biomedical Sciences, Tufts University School of Medicine, Boston, MA, United States

**Keywords:** autism (ASD), Wnt, β-catenin (β-catenin), prefronal cortex, parvalbumin

## Abstract

Multiple human autism risk genes are predicted to converge on the β-catenin (β-cat)/Wnt pathway. However, direct tests to link β-cat up- or down-regulation with autism are largely lacking, and the associated pathophysiological changes are poorly defined. Here we identify excessive β-cat as a risk factor that causes expression changes in several genes relevant to human autism. Our studies utilize mouse lines with β-cat dysregulation in forebrain excitatory neurons, identified as cell types with a convergent expression of autism-linked genes in both human and mouse brains. We show that mice expressing excessive β-cat display behavioral and molecular changes, including decreased social interest, increased repetitive behaviors, reduced parvalbumin and altered expression levels of additional genes identified as potential risk factors for human autism. These behavioral and molecular phenotypes are averted by reducing β-cat in neurons predisposed by gene mutations to express elevated β-cat. Using next-generation sequencing of the prefrontal cortex (PFC), we identify 87 dysregulated genes that are shared between mouse lines with excessive β-cat and autism-like behaviors, but not mouse lines with reduced β-cat and normal social behavior. Our findings provide critical new insights into β-cat, Wnt pathway dysregulation in the brain causing behavioral phenotypes relevant to the disease and the molecular etiology which includes several human autism risk genes.

## Background

Emerging evidence suggests that autism spectrum disorders (ASD) likely stem from combinatorial molecular changes that ultimately impact synaptic and circuit functions. Although genetic studies of families with ASD have identified hundreds of risk genes, typically only one mutated gene has been found per affected individual (O’Roak et al., 2012; Sanders et al., 2015; de la Torre-Ubieta et al., 2016). This disparity highlights the need for defining the associated molecular changes caused by ASD-linked risk factors to gain insights into shared pathologies and thereby identify targets for effective therapeutic intervention. Here, we show that malfunction of β-catenin (β-cat) results in reduced social and increased repetitive behavioral phenotypes and altered expression levels of multiple genes whose human orthologs have been implicated in ASD.

β-cat/Wnt has been defined as one of a small number of convergent pathways whose malfunction may predispose neurons to ASD (Gilman et al., 2011; Iossifov et al., 2012; Neale et al., 2012; O’Roak et al., 2012; Zoghbi and Bear, 2012). Several ASD-linked human gene mutations are predicted to cause up- or down-regulation of β-cat functions, including *ctnnb1* (β-cat) itself (Krumm et al., 2014; Tucci et al., 2014; Krupp et al., 2017), adenomatous polyposis coli (APC; Zhou et al., 2007), *chd8* (Durak et al., 2016), *ank3* (Kloth et al., 2017), *arx* (Cho et al., 2017), *ube3a* (Yi et al., 2017), *prickle1* (Todd and Bassuk, 2018), and *wnt1a* (Martin et al., 2013). However, direct tests for linking β-cat malfunction to autism are largely lacking and the associated pathophysiological changes are poorly defined. Our study provides new insights into the molecular etiologies of autism relevant behavioral phenotypes caused by β-cat dysregulation.

Our previous studies implicate, but do not directly test, excessive β-cat in excitatory neurons as a risk factor for altered social and repetitive behaviors and do not elucidate the associated molecular changes. We have shown that conditional knockout (cKO) of APC, the major negative regulator of β-cat, in mouse forebrain excitatory neurons, causes the expected increases in β-cat and canonical Wnt target gene expression levels, as well as behavioral phenotypes (reduced social interactions, increased repetitive behaviors), cognitive impairments and seizures (Mohn et al., 2014; Pirone et al., 2017) relevant to ASD. However, beyond regulating β-cat levels, APC has other roles critical for neuron maturation and function that are potentially relevant to normal behaviors: including its role in regulating microtubule and actin cytoskeleton dynamics (Zumbrunn et al., 2001; Akiyama and Kawasaki, 2006) and as an mRNA binding protein with several of its targets functioning in brain development (Preitner et al., 2014).

In the present study, we have used new mouse lines with direct genetic manipulation of β-cat in the presence and absence of APC. We show roles of β-cat up- and down-regulation in the brain in causing vs. averting autism relevant social and repetitive behavior phenotypes. Importantly, we identify associated molecular changes, including altered expression levels of several genes linked to autism in humans. Our findings provide critical insights into a molecular etiology of impaired social and repetitive behaviors, with relevance to human ASD-linked genes predicted to dysregulate the β-cat network.

## Materials and Methods

### Animals

APC cKO (*APC*^fl/fl^) mice were generated as previously described (Mohn et al., 2014). β-cat cOE (*ctnnb1*^fl(ex3)/+^; Harada et al., 1999), β-cat cKO (*ctnnb1*^fl/fl^; Wickham et al., 2019), and APC/β-cat cKOs (*APC*^fl/fl^/*ctnnb1*^fl/fl^) mice were generated with the identical CamKIIα-Cre-93 recombinase carrying line (Rios et al., 2001). For all experiments, 2–3 month-old mice of both sexes were used. Littermate controls (Cre negative) were pooled from all lines. Mice of all genotypes were born at Mendelian ratios and showed no deficits in body weight or survivability until the age of testing (although 5% of APC/β-cat cKO mice showed hydrocephaly and were excluded from experiments). All procedures were approved by the Tufts University Institutional Animal Care and Use Committee in accordance with National Institutes of Health guidelines.

### Biochemical Experiments

Western blots and quantitative PCR were performed as previously described (Mohn et al., 2014). Primers for *pvalb* qPCR are: (Fwd) ATCAAGAAGGCGATAGGAGCC (Rev) GGCCAGAAGCGTCTTTGTT. Antibodies used are anti-β-catenin (mouse, 1:2,000, Invitrogen, RRID:AB_2533039), anti-APC (rabbit, 1:1,000, Abcam, RRID:AB_301806), anti-parvalbumin (rabbit, 1:1,000, Swant, RRID:AB_2631173), anti-HSP90 (rabbit, 1:1,000, Cell Signaling, RRID:AB_2233331), and anti-GAPDH (mouse, 1:10,000, Millipore, RRID:AB_2107445).

λ-Phosphatase treatment was performed as previously described (Humrich et al., 2003). Briefly, β-cat cOE and littermate control hippocampi were homogenized in λ-phosphatase buffer containing protease inhibitor cocktail. λ-phosphatase (10,000 U/ml) and magnesium cocktail was added to 100 ug of total protein and incubated at 370C for 1 h (untreated samples did not receive λ-phosphatase but were prepared in the same fashion). Fifty microgram of protein was resolved on a 3–8% gel (Invitrogen) and immunoblots used HSP90 as a loading control.

### Behavioral Assays

Mice were housed on a reversed 12-h light/dark cycle, and handled 5 min daily for a week before behavioral testing. Three chamber test and marble burying were performed as previously described (Mohn et al., 2014). For the repetitive circling assay, mice were removed from their home cage and placed in an empty shoebox cage containing no nestlet. The mice were videotaped for 15 min and were scored by a blinded observer. Criteria for circling behavior was a minimum of two bouts of at least three consecutive, unidirectional, fast circling motions within a restricted area (i.e., circling the outside edge of the cage was not considered circling behavior) during a period of 15 min.

### Spine Density

Fluorescent labeling of neurons for synaptic spine density was done as previously described (Staffend and Meisel, 2011). Neurons were imaged by confocal microscopy (Nikon A1R laser confocal scanning microscope with 63× objective; 3× zoom). Dendritic spines were reconstructed (Imaris software), and spine density was calculated.

### Next-Generation Sequencing

Library preparation, sequencing, and initial expression analysis was performed by the Tufts University Core Facility Genomics lab. Briefly, the quality of input RNA samples was assessed on Advanced Analytical Fragment Analyzer. RNA samples that passed the quality check were used as input for RNA-Seq library preparation using Illumina TruSeq stranded mRNA, following manufacturer instruction. The resultant library was then quantified and pooled equal molar and was sequenced with paired-end 100 bases format on an Illumina HiSeq 2500 using High Output V4 chemistry. Fastq files were generated from raw data using bcl2fastq (Illumina). The fastq files were mapped mouse mm10 reference genome with Tophat2. Normalized read counts were generated with Cufflinks2, and differential expression and hierarchical clustering analyses were performed with Cuffdiff2 and Qlucore Omics Explorer.

### Statistical Analysis

All data are reported as the arithmetic mean ± standard error. Statistical analysis was done using Graphpad Prism 7 and the specific statistical test used are reported in the text and figure legends.

## Results

### New β-cat cOE and APC/β-cat cKO Mouse Lines

We have generated two new mutant mouse lines with dysregulated β-cat during the early postnatal stage of major synaptic differentiation, a critical window of brain development relevant to ASD. We have utilized the CamKIIα Cre driver that is predominantly expressed in forebrain excitatory neurons and fully activated during the first three postnatal weeks in mice (Rios et al., 2001; Pirone et al., 2017) equivalent to the developmental age when glutamatergic neurons exhibit convergent expression of several ASD linked genes in both the human and mouse cortex (Parikshak et al., 2013; Willsey et al., 2013). We have used this CamKIIα-Cre driver to target the same cell types at the same developmental age in all of our mouse lines, including the APC cKO and β-cat cKO lines (Mohn et al., 2014; Wickham et al., 2019).

**Figure 1:**
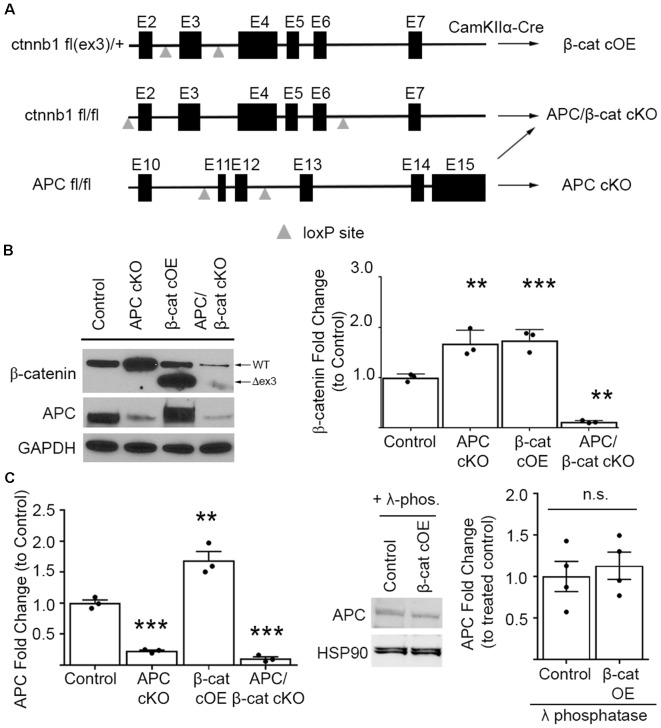
Mouse lines comparing β-cat up- or down-dysregulation. **(A)** Schematic showing the floxed *ctnnb1* and *adenomatous polyposis coli (APC)* genes used to alter β-catenin levels in mice carrying CamKIIα-Cre recombinase. For excessive levels: β-cat cOE-overexpression of stable N-terminally truncated β-cat by deleting the degradation domain, and APC conditional knockout (cKO; Mohn et al., 2014)—deletion of the major negative regulator of β-cat; for reduced levels: APC/β-cat cKO with unstable, rapidly degraded protein products from both genes, and β-cat cKO (Wickham et al., 2019). **(B)** Immunoblot and quantification of β-cat prefrontal cortex (PFC) levels. β-cat increase in β-cat cOEs is comparable to that of APC cKOs, relative to control littermates. APC/β-cat cKOs show drastically reduced β-cat, with residual levels likely from non-CamKIIα expressing cell types (*n* = 3 per genotype. **p < 0.01, ***p < 0.001 to control, *post hoc* Bonferroni corrected t-test). As expected, APC levels in APC cKO and APC/β-cat cKO mice are reduced (*n* = 3 per genotype, **p < 0.01, ***p < 0.001 to control, *post hoc* Bonferroni corrected *t*-test) and, although we observe a mobility shift on the blot consistent with phosphorylation in β-cat cOEs, we observe no change in total APC levels **(C)** compared to control littermates after λ-phosphatase treatment (*n* = 4 per genotype), n.s., non-significant.

To upregulate β-cat in the presence of APC, we conditionally overexpressed (cOE), stabilized, N-terminal truncated β-cat by deleting the degradation domain. We crossed CamKIIα-Cre mice with mice expressing loxP sites flanking exon 3 of the *ctnnb1* (β-cat) gene (Harada et al., 1999; Figure 1A). Exon 3 of *ctnnb1* encodes a domain in the β-cat protein that contains the phosphorylation sites necessary for degradation by the APC/Axin/GSK3B destruction complex.

In our β-cat cOE mice, heterozygous expression of this degradation resistant isoform led to β-cat increases, with total β-cat levels comparable to that of APC cKOs (One-way ANOVA F_(3, 8)_ = 87.39, *p* < 0.001; Figure 1B), allowing us to assess the effects of similarly increased β-cat, in the presence vs. absence of APC, in causing autism relevant behavioral phenotypes. Although we observed what appeared to be an increase in APC in the β-cat cOE cortex by immunoblotting (One-way ANOVA F_(3, 8)_ = 58.61, *p* < 0.0001; Figure 1C), the APC signal has a widespread, and treatment with λ-phosphatase demonstrates that there is no significant difference in the levels of APC between β-cat cOE mice and controls (Student’s t-test, *p* = 0.616; Figure 1C).

Additionally, we generated the double mutant APC/β-cat cKO mouse line to prevent the increase in β-cat in the absence of APC, to test whether the social and repetitive behavioral phenotypes that we observed in APC cKOs is caused by elevated β-cat or APC loss. We crossed the CamKIIα Cre mice with mice expressing loxP sites flanking exon 2 and exon 6 of the *ctnnb1* gene and flanking exon 11 and exon 12 of the *APC* gene (Brault et al., 2001; Gounari et al., 2005; Figure 1A). Cre-mediated recombination results in severely truncated β-cat and APC protein products that are unstable and rapidly degraded. The APC/β-cat cKO mice show large reductions in β-cat, compared to littermate controls, with the slight residual levels most likely due to other cell types that do not express CamKIIα (β-cat: One-way ANOVA F_(3, 8)_ = 87.39, *p* < 0.001; Figure 1B). Similarly, we observe comparable reductions in APC levels between this new line and APC cKO mice relative to controls (APC: One-way ANOVA F_(2, 6)_ = 182.7, *p* < 0.0001; APC cKO: 0.2273 ± 0.0162, *p* < 0.0001 Bonferroni-corrected Student’s t-test; APC/β-cat cKO: 0.1048 ± 0.0303, *p* < 0.0001 Bonferroni-corrected Student’s *t*-test; Figure 1B). We used these new mouse lines to test directly whether excessive β-cat can cause aberrant social and repetitive behavioral phenotypes.

### β-cat cOEs Exhibit Phenotypes Relevant to ASD, But APC/β-cat cKOs Do Not

**Figure 2:**
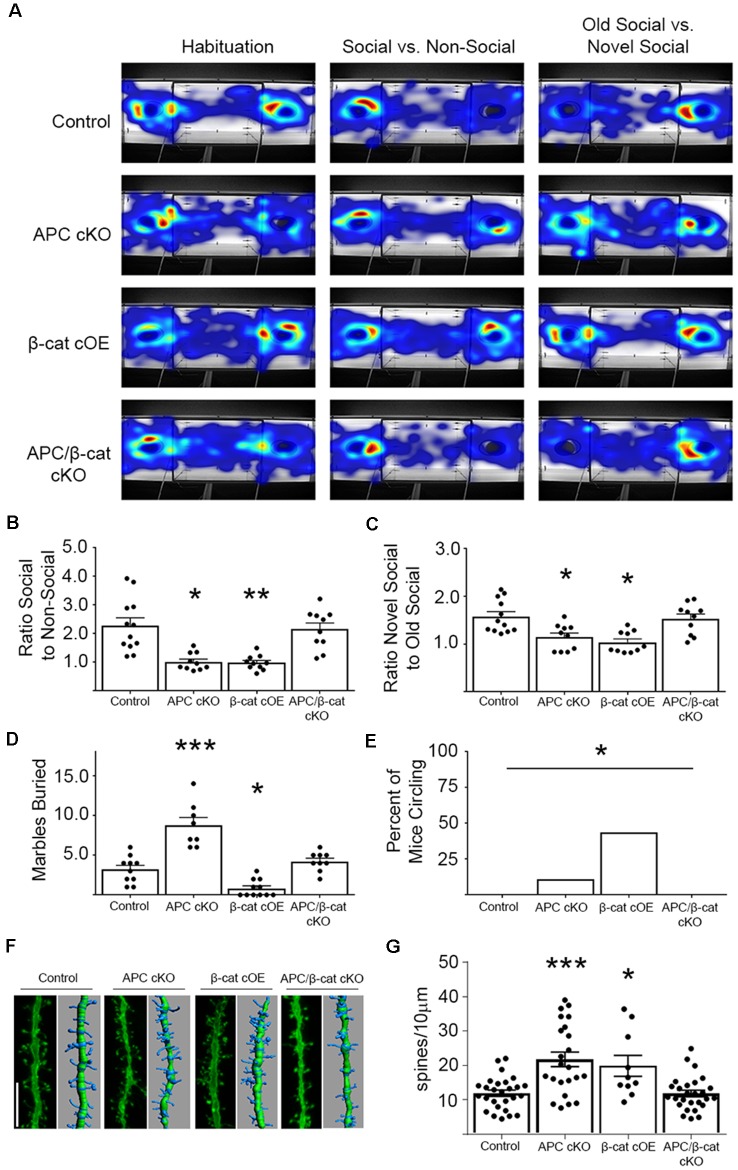
β-cat increases, but not decreases in excitatory neurons, cause behavioral phenotypes relevant to autism spectrum disorders (ASD). **(A)** Representative heat-maps of the mouse models during the habituation, social interaction, and social memory phases of the three-chambered test. **(B)** Both β-cat cOEs and APC cKOs spend a significantly reduced ratio of time interacting with the social cage vs. the empty cage, compared to controls. APC/β-cat cKOs show normal sociability (*n* = 10–12 per genotype; *p < 0.05, **p < 0.01 to control, post-hoc Bonferroni corrected t-test). **(C)** Both β-cat cOEs and APC cKOs spend a reduced percent of time interacting with the novel mouse vs. the familiar mouse, whereas APC/β-cat cKOs interact with the novel mouse more, similar to controls (*p < 0.05 to control, *post hoc* Bonferroni corrected t-test). **(D)** APC cKOs bury significantly more marbles than controls in the marble burying assay. This repetitive behavior is prevented in APC/β-cat cKOs (*n* = 8–11 per genotype; *p < 0.05, ***p < 0.001, post-hoc Bonferroni corrected t-test). β-cat cOEs bury significantly fewer marbles than controls, and exhibit **(E)** repetitive circling behavior (*n* = 11–18 per genotype; *p < 0.05, Chi-squared test). **(F)** Representative images and Imaris reconstructions of the proximal apical dendrite of layer V cortical neurons from the various mouse models. **(G)** Mice with elevated levels of β-cat (APC cKO, β-cat cOE) show increased dendritic spine density (*n* = 3–5 animals per genotype, 3–8 neurons per animal; *p < 0.05, ***p < 0.001, *post hoc* Bonferroni corrected *t*-test) that is corrected in APC/β-cat cKO mice with low levels of β-cat.

Using the classic three-chamber assay (Crawley, 2007), we tested for altered social interactions in the β-cat cOEs (elevated β-cat, normal APC levels) and APC/β-cat cKOs (reduced β-cat, reduced APC), compared to their control littermates and to APC cKOs (elevated β-cat, reduced APC). Relative to control littermates, β-cat cOEs displayed reduced social interest, measured as the ratio of time spent interacting with the novel mouse cage vs. the empty cage (One-way ANOVA, F_(3, 38)_ = 13.17, *p* < 0.001; Figures 2A,B). Distance traveled and velocity was normal (calculated during the habituation phase), eliminating motor deficits as a potential confound (One-way ANOVA F_(3, 38)_ = 0.4467, *p* = 0.7211, data not shown). The reduced social interactions of β-cat cOEs resembles that seen in APC cKOs (Figures 2A,B). In contrast, APC/β-cat cKOs demonstrated normal social interest (Figures 2A,B) suggesting that the aberrant social behavior of APC cKOs is averted by preventing elevated β-cat in neurons predisposed to excessive β-cat by APC loss. Similarly, β-cat cKOs (low β-cat, normal APC levels) show that β-cat down-regulation in the excitatory neurons, using the same CamKIIα-Cre driver did not affect their social behavior, relative to control littermates (Wickham et al., 2019).

Next, we tested for deficits in social memory using a novel vs. familiar mouse in the three-chamber paradigm. β-cat cOEs spent a significantly reduced ratio of time interacting with the novel mouse cage, relative to the familiar mouse cage, suggesting reduced social memory (One-way ANOVA F_(5, 50)_ = 4.015, *p* = 0.0039; Figures 2A,C). This resembles the deficiencies in the social memory of APC cKOs (Figures 2A,C). In contrast, APC/β-cat cKOs displayed increased interactions with the novel mouse cage, similar to control littermates, averting the reduced social interest phenotype of APC cKOs alone (Figures 2A,C).

We also tested for repetitive behaviors, using marble burying, a repetitive digging task (Thomas et al., 2009). Whereas APC cKOs buried significantly more marbles than control littermates (Figure 2D), the β-cat cOEs buried fewer marbles than their littermate controls (One-way ANOVA F_(3, 34)_ = 32.43, *p* < 0.0001; Figure 2D). Observing their behavior showed that β-cat cOEs spent much of the time unidirectionally circling in the marble-containing novel environment, suggesting repetitive stereotypy behavior (Chi-squared 14.02, *df* = 3, *p* = 0.029; Figure 2E). The APC/β-cat cKOs buried a comparable number of marbles to control littermates and did not circle, suggesting that lowering β-cat prevents the autism relevant repetitive behavior phenotype seen in APC cKOs. Similarly, mice with β-cat cKO alone displayed normal behavior in the marble-burying assay (Wickham et al., 2019).

Studies of post-mortem brains from autistic patients show that dendritic complexity and spine density are commonly altered in the disease (Hutsler and Zhang, 2010; Tang et al., 2014; Weir et al., 2018). Our previous studies in APC cKO mice showed increases in cortical spine density concurrent with the ASD relevant behavioral phenotypes (Mohn et al., 2014). To assess whether spine density is similarly altered in the new β-cat cOE mouse line with high β-cat in the presence of APC, we employed gene-gun labeling of individual neurons in brain slices from the different mutant mouse lines followed by confocal microscopy and Imaris reconstruction analysis (Figure 2F). Similar to APC cKOs, β-cat cOEs showed an increase in dendritic spine density, measured on the 1^st^ branch of the apical dendrite of layer V cortical neurons (One-way ANOVA F_(3, 83)_ = 11.48, *p* < 0.0001; Figure 2G). In contrast, APC/β-cat cKO mice show no significant difference in spine density, relative to control mice. These data suggest that high-levels of β-cat in glutamatergic neurons of the forebrain *in vivo* result in increased spine density, similar to what has been observed in primary cultures of hippocampal neurons (Murase et al., 2002).

### Elevated β-cat Causes Altered Expression of Several Genes Linked to Human ASD

**Figure 3:**
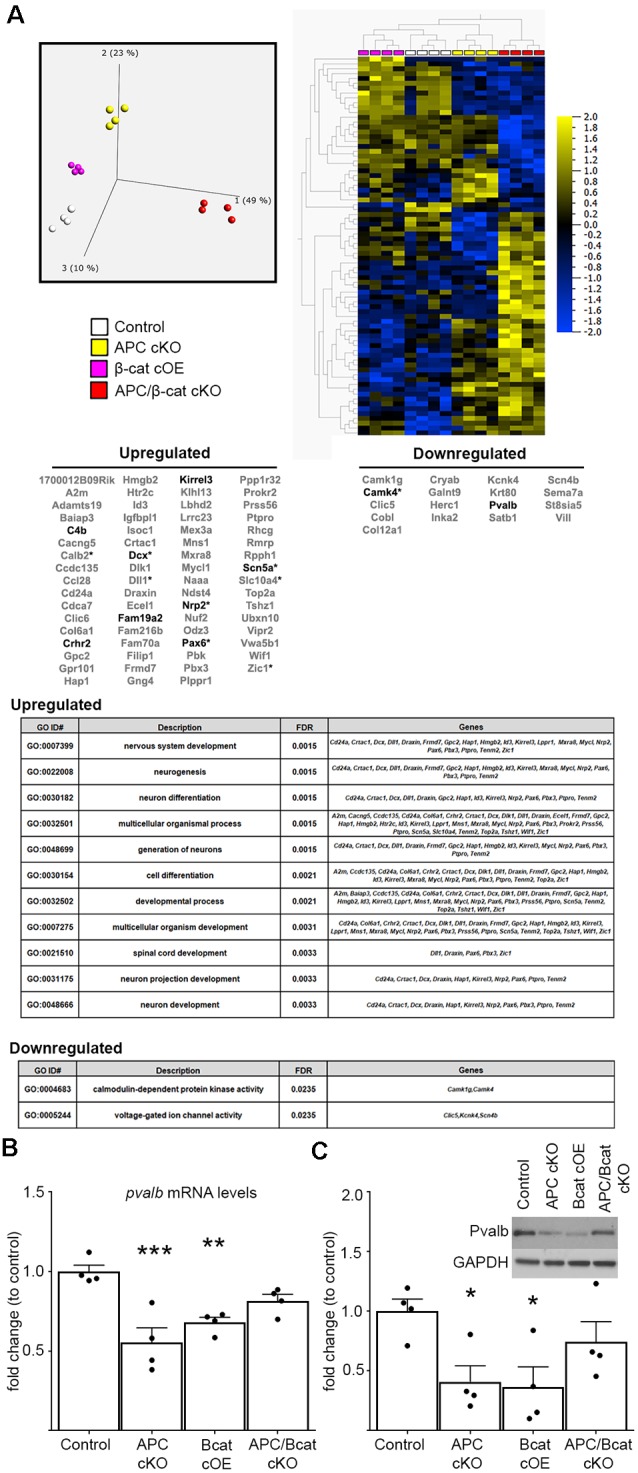
Elevated β-cat causes altered expression of multiple autism-linked genes. **(A)** Next-generation data sets (*n* = 4 per genotype) were subject to primary component analysis and hierarchical clustering. The PFC shows 87 dysregulated genes (70 upregulated, 17 downregulated) shared between the two elevated β-cat models that exhibit altered social and repetitive behaviors, but not the reduced β-cat model that does not. Nine of the dysregulated genes are canonical Wnt target genes (asterisks). Ten are associated with human ASD (SFARI AutDB, bolded), and gene ontology analysis shows altered functions relevant to ASD pathology. Decreases in one of the identified ASD-linked genes, *pvalb*, at both **(B)** mRNA (qPCR) and **(C)** protein (immunoblot) levels in PFC of β-cat cOEs and APC cKOs, but normal levels in APC/β-cat cKOs (*n* = 4 per genotype, *p < 0.05, **p < 0.01, ***p < 0.0001, Sidak-Bonferroni corrected Student’s *t*-test).

To begin to identify the molecular etiology of aberrant social and repetitive behaviors caused by increased β-cat in our mice, we employed unbiased next-generation sequencing of RNA from the prefrontal cortex (PFC)—a brain region associated with social behavior and implicated in ASD in human studies (Hashemi et al., 2017; Selimbeyoglu et al., 2017; Brumback et al., 2018; Carvalho Pereira et al., 2018; Lazaro et al., 2019). We compared mice with elevated β-cat and altered social and repetitive behaviors (β-cat cOEs, APC cKOs), mice with reduced β-cat that do not display the phenotypes (APC/β-cat cKOs) and control littermates. We found 87 dysregulated genes (70 increased, 17 decreased) that are shared between the elevated β-cat mouse lines, but not the reduced β-cat line (Figure 3A; primary component analysis ANOVA *p* = 0.001, *q* = 0.298). Nine of the dysregulated genes are canonical Wnt targets (Hödar et al., 2010; Wisniewska et al., 2012; Figure 3). Gene Ontology analysis for the up- and down-regulated gene sets show enrichment for several GO terms relevant to circuit malformations: *neuron projection development* (*q* = 0.0033) and *neuron differentiation* (*q* = 0.0015).

Importantly, 10 of the 87 dysregulated genes are annotated in the SFARI AutDB database (*crhr2, scn5a, pax6, c4b, dcx, kirrel3, fam19a2, nrp2, camk4, pvalb*), displaying a significant overrepresentation of gene changes in our models potentially linked to ASD (One-sided Fisher’s exact test, *p* = 0.001). These genes function in neuron migration, cytoskeleton dynamics, cell adhesion, axon guidance, and neural activity. Our findings suggest that β-cat networks, dysregulated by excessive β-cat in excitatory neurons, leads to aberrant expression levels of multiple genes implicated in human autism.

From these 10 ASD-linked genes, we have focused initially on the downregulated gene, *pvalb*, encoding the calcium-binding protein, parvalbumin, in fast-spiking interneurons. Excitatory/inhibitory imbalance in the PFC has been shown to alter social behaviors (Yizhar et al., 2011), and our previous study of the APC cKO mouse shows a reduced number of parvalbumin-positive cells in the medial PFC, increased c-fos in excitatory neurons in the infralimbic subregion in response to a novel social stimulus and increased mEPSC frequency (Pirone et al., 2018). qPCR and immunoblots show reductions in *pvalb* mRNA (One-way ANOVA F_(3, 12)_ = 11.07, *p* = 0.0009; Figure 3B) and protein levels (One-way ANOVA F_(3, 12)_ = 4.1742, *p* = 0.0306; Figure 3C) in the PFC of both mouse lines with elevated β-cat. In contrast, PFC parvalbumin protein and mRNA levels are normal in APC/β-cat cKOs that do not exhibit the aberrant social and repetitive behaviors (Figures 3B,C). Consistent with our results, reduced parvalbumin levels in the PFC have been associated with altered social behaviors in both human and mouse studies (Wöhr et al., 2015; Filice et al., 2016; Hashemi et al., 2017).

## Discussion

Our major findings are that excessive β-cat leads to decreased social interest and increased repetitive behaviors and aberrant expression of multiple genes that have been implicated in human ASD and play roles in synaptic function and circuit connections in the brain. Decreasing β-cat in neurons predisposed by gene mutations to express excessive β-cat averts these phenotypes. Our data are elucidating how autism-linked human genes that converge on the β-cat network may incline neurons to disease.

Our APC and β-cat mutant mouse lines are experimentally amenable models of the mammalian brain. Although these conditional mutants are not direct models of disease-linked human gene mutations, they are valuable tools to elucidate the pathophysiological consequences of aberrant β-cat levels in neurons *in vivo*. Importantly, our β-cat genetic manipulations target glutamatergic neurons, at a relevant developmental age, as it coincides with the stage when cortical glutamatergic neurons of both the human and mouse brain display convergent expression of several ASD and ID linked genes (Parikshak et al., 2013; Willsey et al., 2013). Thus the time-frame of our β-cat genetic manipulations may target a critical developmental window. Conditional manipulation of β-cat and APC gene expression during synaptic differentiation, rather than earlier or later, is necessary to define behavioral and cognitive phenotypes. Global nulls lead to embryonic lethality (Haegel et al., 1995; Moser et al., 1995; Huelsken et al., 2000). Conditional deletion and overexpression in progenitor cells causes severe brain malformation (Haegel et al., 1995; Moser et al., 1995; Brault et al., 2001; Chenn and Walsh, 2002, 2003; Gao et al., 2007; Grigoryan et al., 2008; Maguschak and Ressler, 2008; Ivaniutsin et al., 2009).

Our findings provide the first direct *in vivo* evidence, to our knowledge, that excessive levels of β-cat can lead to social deficits and increased repetitive behaviors. While most mutations in human *ctnnb1* (β-cat) gene result in loss of function of the protein (Krumm et al., 2014; Krupp et al., 2017), work by us and others suggests that maintenance of the proper levels of β-cat is critical for normal behavior and may have cell type-specific repercussions. This is highlighted by the fact we do not observe these behavioral deficits in our mice with low levels of β-cat in excitatory neurons, but social deficits have been observed in mice with deletion of β-cat in parvalbumin interneurons (Dong et al., 2016). Further, mutations in several other ASD-linked human genes are predicted to cause up- or down-regulation of β-cat functions.

Intriguingly, although the β-cat cOE and APC cKO mice show similar increases in β-cat protein levels, we note some differences in their behavioral phenotypes. While social interest is reduced to a similar extent, β-cat cOEs exhibit increased circling and a reduction in marble-burying activity relative to APC cKOs. Further, β-cat cOEs show hyperphosphorylation of APC, which may play a role in the behavioral differences. As APC is a large protein with 180 putative phosphorylation sites, further studies will be needed to test whether APC function is altered and its potential contribution to the divergent phenotypes. Additionally, APC loss in the APC cKOs may also impact the behavioral phenotypes compared to the β-cat cOEs.

The ability of this one dysregulated protein to cause a cascade of molecular changes that impact behavior likely derives from its role in two core pathways- the cadherin synaptic adhesion complex and canonical Wnt signal transduction. β-cat links the synaptic adhesion complex to the submembranous actin cytoskeleton, thereby stabilizing the synapse (Knudsen et al., 1995; Uchida et al., 1996; Yu and Malenka, 2004; Brigidi and Bamji, 2011). Additionally, β-cat binds directly to key postsynaptic scaffolds, the synaptic scaffolding cell adhesion molecule (S-SCAM/Magi2) and APC, that bring together other adhesion proteins, glutamate receptors and signaling molecules that impact synapse maturation and function (Nishimura et al., 2002; Rosenberg et al., 2010; Mohn et al., 2014). In the canonical Wnt signaling pathway, β-cat functions as a transcription co-activator with TCF/LEF to mediate Wnt responsive gene expression (Clevers and Nusse, 2012). Several studies show that manipulating cadherin and Wnt signaling in the brain alters axon guidance cues, synapse maturation, density and plasticity, and network connectivity (Uchida et al., 1996; Mysore et al., 2007; Brigidi and Bamji, 2011; Park and Shen, 2012; Salinas, 2012; Rosso and Inestrosa, 2013).

Studies of cultured hippocampal neurons show that excessive β-cat increases dendritic branching, spine density and synaptic function (mEPSC frequency), suggesting the potential for excitability imbalance (Murase et al., 2002; Yu and Malenka, 2003, 2004; Okuda et al., 2007). Increased neural activity by optogenetic activation of glutamatergic pyramidal neurons in the PFC of wild-type mice is sufficient to cause reduced social interest (Yizhar et al., 2011). Both of our mouse lines with elevated β-cat, β-cat cOEs, and APC cKOs, display increased dendritic spine density and reductions in parvalbumin mRNA and protein levels. Further, APC cKOs exhibit increased excitation of pyramidal neurons in the medial PFC when presented with a novel social stimulus (Pirone et al., 2018). Preventing the increase in β-cat in APC cKOs (APC/β-cat cKOs) averts the reductions in parvalbumin and corrects the social and repetitive behavioral phenotypes. The decreases in parvalbumin in β-cat cOEs and APC cKOs are likely caused by non-cell autonomous changes in the cellular microenvironment as reporter studies of CamKIIα Cre mice show that it is not expressed in these interneurons (Rios et al., 2001; Pirone et al., 2017). Parvalbumin interneuron specification, including migration, localization, maturation and synaptogenesis, are known to be regulated by both intrinsic and cellular microenvironment signaling (Wamsley and Fishell, 2017; Loo et al., 2019). Future studies are needed to identify the signaling factors responsible for the reductions in parvalbumin. It is also important to assess the other molecular changes found in the β-cat cOEs and APC cKOs to elucidate their impact on excitatory and inhibitory synaptic and circuit functions that are critical for normal behavior.

Our findings define a novel role for β-cat by showing that its dysregulation leads to altered expression of several genes linked to autism in humans. We provide new insights into molecular changes caused by malfunction of β-cat, one of a small number of convergent targets identified in human ASD. Elucidating the molecular etiologies of ASD is essential for identifying shared pathological changes that may be root causes and potential targets for effective therapeutic intervention.

## Data Availability Statement

The datasets analyzed in this study can be found on the GEO Database at https://www.ncbi.nlm.nih.gov/geo/query/acc.cgi, under accession no: GSE147034.

## Ethics Statement

All procedures involving animals were approved by the Tufts University Institutional Animal Care and Use Committee under the National Institutes of Health guidelines.

## Author Contributions

JA and MJ designed the research studies. JA performed behavioral and biochemical research and analyzed data. AP performed spine analysis studies. JA and MJ wrote the manuscript.

## Conflict of Interest

The authors declare that the research was conducted in the absence of any commercial or financial relationships that could be construed as a potential conflict of interest.
